# *Heteroctenus junceus* Scorpion Venom Modulates the Concentration of Pro-Inflammatory Cytokines in F3II Tumor Cells

**DOI:** 10.3390/life13122287

**Published:** 2023-11-30

**Authors:** Arianna Yglesias-Rivera, Hermis Sánchez-Rodríguez, Carmen Soto-Febles, Lianet Monzote

**Affiliations:** 1 Research Department, Laboratories of Biopharmaceutical and Chemistry Productions (LABIOFAM), Ave. Independencia Km 16 1/2, Santiago de las Vegas, Boyeros, La Habana 10800, Cuba; 2 Microbiology Department, Institute of Tropical Medicine “Pedro Kouri”, Autopista Novia del Mediodía Km 6 1/2, La Lisa, La Habana 17100, Cuba; hermis@ipk.sld.cu; 3 Center for Protein Studies, Biology Faculty, University of Havana, Calle 25 Entre J e I, # 455, Plaza de la Revolución, La Habana 10400, Cuba; carmensoto@fbio.uh.cu

**Keywords:** *Heteroctenus junceus* scorpion venom, pro-inflammatory cytokines, F3II, antitumoral effect

## Abstract

The ability of *Heteroctenus junceus* scorpion venom to modulate the concentration of cytokines related to its antitumoral effect is unknown. F3II cells were treated with ¼ IC_50_, ½ IC_50_ and the IC_50_ of *H. junceus* scorpion venom. Tumor growth kinetics in F3II-bearing mice were evaluated after 24 days of oral administration of venom doses. The effect of tumor lysates on F3II cell viability was evaluated by MTT assay, while cytokines present in each sample were determined by ELISA. In supernatant, *H. junceus* scorpion venom decreased the concentration of IL-6 (*p* < 0.001), IFN-γ (*p* < 0.001), IL-1β (*p* < 0.01); meanwhile IL-12 (*p* < 0.001) and TNF-α (*p* < 0.001) levels increased significantly, according to the concentration and the time of incubation. *Heteroctenus junceus* scorpion venom effectively inhibits in vivo tumor progression. In the sera, a significant decrease was observed in TNF-α levels (*p* < 0.05). In tumor lysates, IL-6 decreased significantly in the groups treated with 12.5 mg/kg (*p* < 0.001) and 25 mg/kg (*p* < 0.05). *Heteroctenus junceus* scorpion venom is capable of modulating other proinflammatory and protumoral cytokines involved in the inflammation associated with cancer.

## 1. Introduction

Cancer is one of the diseases with highest impact worldwide [1]. In both sexes, breast cancer has surpassed lung cancer as the most commonly diagnosed cancer, followed by lung, colorectal, prostate, and stomach cancers [2]. Lung cancer has the highest mortality rate, followed by breast, colorectal, liver, stomach, prostate, and uterine cervical cancer [2]. In Cuba, cancer is the second leading cause of death. Over the last 20 years, the most common causes of cancer death in Cuba have been lung, prostate, and colon cancers in men and lung, breast, and colon cancers in women [3].

Conventional cancer treatment includes three basic methods: surgery, radiotherapy, and chemotherapy [4]. However, its toxic side effects, as well as the resistance it generates, have limited its application in the clinic and have promoted the development of new therapeutic strategies against cancer [5]. In various clinical and preclinical studies, natural products have shown antitumor and antimetastatic potential [6] by modulating the immune response as well as various cellular processes such as signal transduction, cell division, and apoptosis [7,8].

The relationship between cancer cells or tumors with the human immune system is a complex and dynamic process, where proinflammatory cytokines and other direct effectors of the immune response are present [9]. Cytokines, such as TNF-α, IL-6 and IL-1β, are essential in the initiation of chronic inflammation and play a crucial role in the activation of the NF-κB signaling pathway [10]. The network of cytokines secreted by various tumors is dominated by inflammatory cytokines, growth factors, and chemokines [11]. There is now evidence that inflammatory cytokines and chemokines, which may be produced by tumor cells and/or tumor-associated leukocytes and platelets, may directly contribute to malignant progression [12].

Scorpion venom used in traditional medicine for therapeutic purposes has shown antitumor potential. The venom of the scorpion *Heteroctenus junceus* (*H. junceus*) Herbst 1800 (previously known as *Rhopalurus junceus*) [13] is an endemic species of Cuba that belongs to the Buthidae family, and has displayed anti-inflammatory, analgesic, and antitumor properties [14,15]. From a scientific point of view, previous in vitro studies have shown that this venom exerts a selective cytotoxic effect against tumors of epithelial origin such as: lung (A549, NCI-H292), breast (MDA-MB-231, MDA-MB-468), cervix (HeLa, SIHA), larynx (Hep-2), and colorectal (HT29); without affecting normal cells: MRC-5, Vero and MDCK [14]. In addition, this scorpion venom induced a significant cytotoxic effect in F3II cells and a significant inhibition of tumor progression in F3II-bearing mice in a dose-dependent manner by the intraperitoneal route [16]. However, different aspects of its action at the molecular level on tumor cells, antitumor effect by oral route, and its immunomodulatory capacity are still unknown, which is addressed in this study.

## 2. Materials and Methods

### 2.1. Venom Source and Extraction

*Heteroctenus junceus* scorpions were maintained in individual plastic cages in the laboratories belonging to The Entrepreneurial Group of Biopharmaceutical and Chemistry Productions (LABIOFAM, Havana, Cuba). Utilizing electrical stimulation, scorpions kept alive in the laboratory were milked for venom extraction, specifically with the help of pointed electrode, electric current (25 V) [17] was applied at the base of telson for 5 s to shock the scorpions until the venom was released. The venom was dissolved in distilled water and centrifuged at 10,000× *g* for 15 min. The supernatant was filtered using 0.2 μm syringe filters and stored at −20 °C until used. The protein concentration was calculated using the modified Lowry’s method [18]. The biochemical and molecular characterization of *H. junceus* scorpion venom was previously described by García-Gómez et al. (2011) [19] (Figure S1) and Rodríguez-Ravelo et al. (2015) [20] (Table S1).

### 2.2. Cell Line and Culture

The mouse F3II mammary adenocarcinoma (metastatic from a spontaneous tumor in BALB/c mice) was donated by Center of Molecular Immunology (Havana, Cuba). The cells were grown and maintained in DMEM (Sigma, St. Louis, MO, USA), supplemented with 10% of fetal bovine serum (FBS) heat-inactivated purchased from Hyclone (Logan, UT, USA), 2 mM L-glutamine, and 80 μg/mL gentamicin (Sigma, St. Louis, MO, USA). Cells were routinely passaged using 0.25% trypsin (Sigma, St. Louis, MO, USA) containing 0.02% EDTA (Sigma, St. Louis, MO, USA).

### 2.3. Animals and Management Scheme

Adult male BALB/c mice obtained from the National Center for Laboratory Animal Breeding (CENPALAB, Havana, Cuba) were used. They were housed in plastic cages under standard conditions; food and water were administered ad libitum. The experimental procedure using animals was approved by the Institutional Committee for Care and Use of Laboratory Animals (Protocol 2013/3), performed in accordance with the EU Directive 2010/63/EU for animal experiments and considering the recommendations of the Guide for the Welfare and use of Animals in Cancer Research [21].

### 2.4. Culture Supernatant, Sera and Tumor Lysate

F3II tumor cells were cultured at 1 × 10^5^ cells/well for 24 h in 24-well plates in a 5% CO_2_ atmosphere at 37 °C. After this time, the cells were treated with 0.25; 0.5 and 1 mg/mL of *H. junceus* scorpion venom for 24 and 48 h. Subsequently, the supernatants were extracted, the total protein concentration was determined, and its concentration was adjusted to 100 μg/mL in DMEM supplemented with 10% of FBS.

F3II cells (2 × 10^5^ cells/0.2 mL DMEM) were injected into the subcutis of the right flank of BALB/c mice. After 11 days of tumor implementation, mice were distributed in 4 groups of 7 mice each and 6.25; 12.5 and 25 mg/kg of scorpion’s venom were administered for 24 consecutive days by oral route. Twenty-four hours after the last oral administration of *H. junceus* scorpion venom, three mice were randomly selected from each experimental group and euthanized by cervical dislocation. Blood samples were taken using a capillary in the ocular plexus, incubated for 30 min at 37 °C, 1 h at 4 °C, and centrifuged at 660× *g* for 10 min to obtain serum. In parallel, tumors were extracted from these mice, individually macerated in a lysis buffer solution (1% protease inhibitor cocktail; Sigma-Aldrich, St. Louis, MO, USA) and 1 M Tris, 0.5 M EDTA, 1 M NaCl, 100 mM DTT and NP-40 (BD Biosciences, San Diego, CA, USA). Each macerate was centrifuged at 1540× *g* for 30 min and the supernatant was selected.

In each case, a total of three replicates were performed and the protein concentration was determined by the modified Lowry method [18].

### 2.5. Antitumoral Effect of H. junceus Scorpion Venom in F3II Bearing BALB/c Mice

To evaluate the effect of scorpion venom on local tumor growth, F3II cells (2 × 10^5^ cells/0.2 mL DMEM) were injected into the subcutis of the right flank of BALB/c mice. After 11 days of tumor implementation, mice were distributed in 5 groups of 7 mice each, and 3 scorpion venom doses were orally administered (6.25; 12.5; 25 mg/kg) for 24 consecutive days. The control group was treated with 200 µL of phosphate-buffered saline (PBS, pH 7.4). The conventional cytostatic cisplatin (CDDP; AICA Laboratories Company, BioCubaFarma, Havana, Cuba) was used as a positive control and a dose of 4 mg/kg was administered three times intraperitoneally on days 11, 18, and 25 post-implementation (pi) of tumor cells. During the following 35 days pi, tumor size was measured with a caliper in two dimensions (width and length) twice a week, and volume was calculated by the following formula: tumor volume = [length (mm) × width^2^ (mm)] × 0.5. After 24 h of treatment with the last dose of scorpion venom of *H. junceus* (36 days pi), three mice were randomly selected from each experimental group and sacrificed by cervical dislocation. Tumors were excised and their weight was determined using a Sartorius balance (Goettingen, Germany). In total, three experiments were performed.

### 2.6. In Vitro Cell Viability Assay (MTT Assay)

F3II cells were seeded in 96-well plates (1 × 10^4^ cell/well) in 50 μL of medium/well in 96-well culture plates and treated with the previously obtained tumor lysates dissolved in culture medium (DMEM supplemented with 2 mM glutamine and nonessential amino acids, 10% of FBS and penicillin-streptomycin 100 IU/mL-100 µg/mL) and added at a final concentration of 100 µg/mL. Untreated cells represent 100% of cell growth and were used as negative control. The plates were additionally incubated at 37 °C and 5% CO_2_ for 72 h. Afterwards, 10 μL of 5 mg/mL of sterile 3-[4,5-dimethylth-iazol-2-yl]-2,5-diphenyl tetrazoliumbromide (MTT, Sigma, St. Louis, MO, USA) was added per well and incubated for an additional 3 h [22]. The supernatant was removed and 150 μL DMSO was added per well. The absorbance was measured with a microplate reader (ELISA MRX Revelation Dynex Technologies, Woonsocket, RI, USA) at 560 nm and 630 nm as reference. Percentage of cell viability was expressed using the following formula: % viability = A_560–630nm_ of treated cells/A_560–630nm_ of negative control cells × 100%.

### 2.7. Cytokine Measurement

Culture supernatant, sera from BALB/c mice implanted with F3II, and tumor lysates were collected, and the presence of cytokines TNF-α (Cat.# 554589), IL-6 (Cat.# 554582), IL-2 (Cat.# 550069), IL-1β (Cat.# 554577), IFN-γ (Cat.# 554587), IL-12 (p70, Cat.# 554592), IL-4 (Cat.# 550067), and IL-10 (Cat.# 550070) were measured by an ELISA assay using pairs of monoclonal antibodies (BD OptEIATM; BD Biosciences, San Diego, CA, USA). The procedure was performed according to the manufacturer’s instructions. The absorbance was determined at 450 nm and 570 nm as reference in an ELISA reader. Each measurement was performed in duplicate, the concentrations of each cytokine in pg/mL were determined by extrapolation of the standard curves evaluated in parallel, and the results were expressed or represented as the average of the values obtained ± the standard deviation (SD).

### 2.8. Statistical Analysis

In the study comparing the tumor volume at different times among the untreated control with each concentration of scorpion venom, a repeated measures two-way ANOVA test was performed, and the Dunnett test was used as a multiple comparison method. This comparison was conducted using the statistical software R version 4.1.1 (R Foundation for Statistical Computing, Vienna, Austria). The one-way ANOVA method was applied in rest of studies, and multiple comparisons were performed using Tukey’s test, using the GraphPad Prism version 5.01 program (GraphPad Software Inc., San Diego, CA, USA). In all cases, differences were considered significant when *p* < 0.05.

## 3. Results

### 3.1. Antitumoral Effect of H. junceus Scorpion Venom in F3II Bearing-Mice

**Figure 1:**
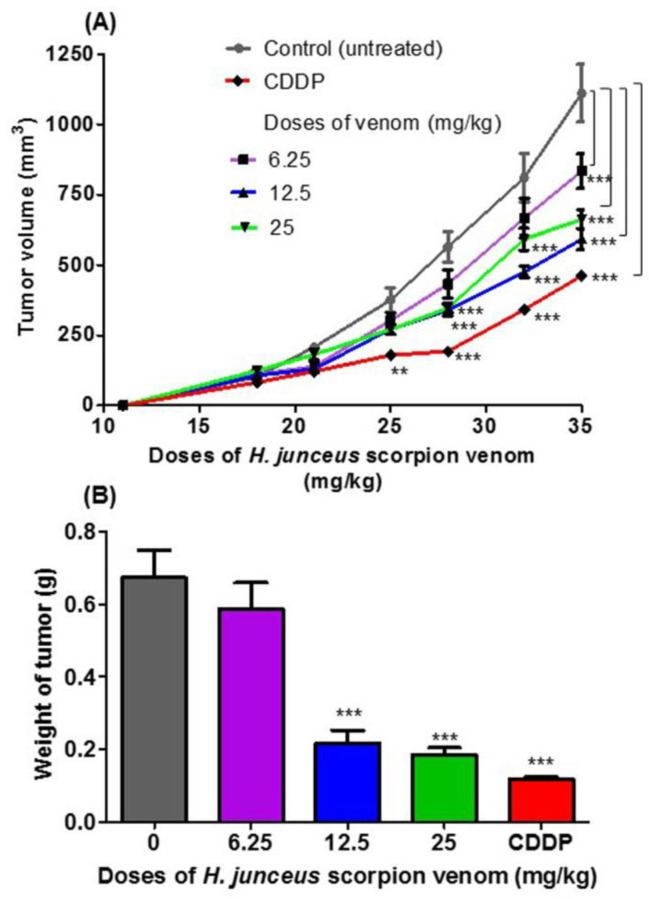
Antitumor effect of *Heteroctenus junceus* (*H. junceus*) scorpion venom on F3II murine mammary adenocarcinoma. (**A**) Tumor volume progression. (**B**) Weight of excised tumor. The experimental groups were inoculated subcutaneously with the F3II tumor cell 1line. Tumor growth was determined during the 35 days after implantation. Oral venom administration of three doses of *H. junceus* scorpion venom began 10 days after tumor implantation, which is indicated on the graph by an arrow. Tumor volume was determined as described in the Section 2. Weight of the F3II tumors extracted 24 h after the last administration of the *H. junceus* scorpion venom. Values represent the mean ± SD of three experiments performed independently with *n* = 10 animals per experimental group each one. Significant differences with respect to F3II-bearing mice control group, ** *p* < 0.01, *** *p* < 0.001, according to repeated measures two-way ANOVA, post-test: Dunnett (**A**); and *** *p* < 0.001, according to one-way ANOVA and Tukey’s multiple comparison test (**B**).

No deaths of animals were observed during the experiment, and the clinical status of the mice was favorable, except in the group treated with cisplatin (CDDP), in which weight loss was observed in all BALB/c mice after the last administration. At the start of the treatment, tumor was palpated in all included animals. In Figure 1A, the effect of oral administration of *H. junceus* scorpion venom on F3II tumor growth is shown. At the beginning of the treatment, the incidence of the tumor was 100% in all the experimental groups. The lowest dose tested, 6.25 mg/kg for 24 days orally, did not affect tumor growth compared with control group (*p* > 0.05). However, 35 days pi, statistically significant differences (*p* < 0.001) were observed in tumor volume values. The experimental groups treated with scorpion venom at doses of 12.5 mg/kg and 25 mg/kg showed a delay in tumor progression when compared to the control group, which was statistically significant after 28 days pi (*p* < 0.001). However, oral administration of venom for 24 days at a rate of once daily administration showed that the dose of 12.5 mg/kg had the maximum effect on tumor growth. The positive control used in the experimentation was the conventional cytostatic CDDP, which demonstrated a statistically significant decrease in tumor volume for days 25 pi (*p* < 0.01) and days 28, 32, and 35 pi (*p* < 0.001) with respect to the control group. In the weight of the F3II tumors extracted 24 h after the end of the treatment (36 days pi), a statistically significant reduction (*p* < 0.01) was observed with respect to the control group for the doses of 12.5 and 25 mg/kg (Figure 1B).

### 3.2. Effect of Tumor Lysates on the Viability of F3II Tumor Cells

**Figure 2:**
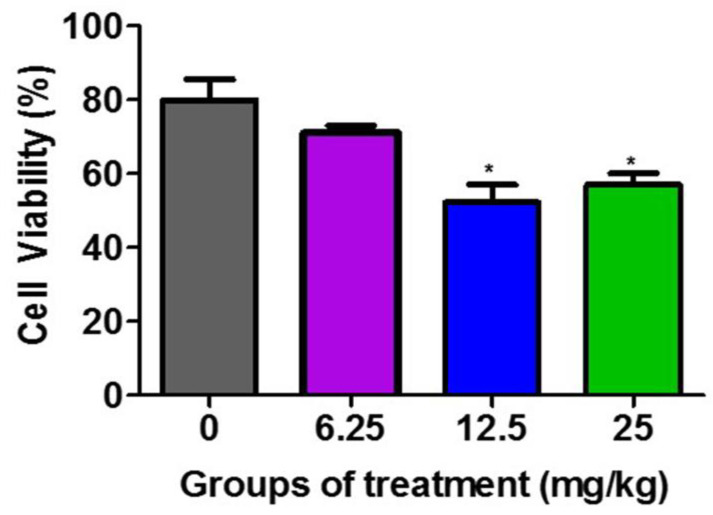
Effect of F3II tumor lysates on the viability of F3II tumor line. The murine metastatic breast tumor line F3II was incubated for 72 h with 100 µg/mL of the obtained tumor lysates from the extraction of the F3II tumor implanted in BALB/c mice treated with 6.25, 12.5 mg/kg, and 25 mg/kg of *H. junceus* scorpion venom. Cell viability was determined by the MTT assay. Values represent the mean ± SD of five replicates from 3 independent experiments. Significant differences * *p* < 0.05, with respect to the cells without scorpion venom treatment, according to one-way ANOVA and Tukey’s post-test.

Figure 2 demonstrates that the F3II tumor lysates from the groups treated with 12.5 mg/kg and 25 mg/kg of the *H. junceus* venom significantly (*p* < 0.001) reduced the viability of the F3II tumor line, compared to the untreated control group. These differences between the percentage of viable cells between the lysates of the groups treated with 12.5 mg/kg and 25 mg/kg with respect to the untreated control is selective over the F3II tumor line. 

### 3.3. Cytokine Levels in F3II Supernatant

**Figure 3:**
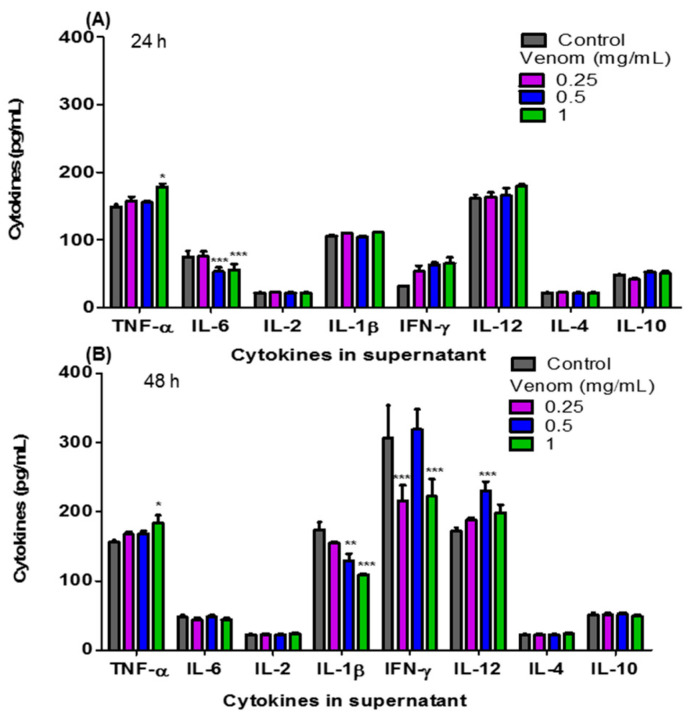
Concentrations of the pro-inflammatory and anti-inflammatory cytokines present in the culture supernatant of F3II treated with *H. junceus* scorpion venom. The concentration values in pg/mL of TNF-α, IL-6, IL-2, IL-1β, IFN-γ, IL-12, IL-4 and IL-10, were determined through an Immunoenzyme Assay using a commercial kit (BD OptEIA™; BD Biosciences, San Diego, CA, USA) after 24 (**A**) and 48 (**B**) hours of incubation with ¼ IC_50_, ½ IC_50_, and the IC_50_ of *H. junceus* scorpion venom. IC_50_: concentration values that reduce 50% of cell viability. Results are expressed as mean ± SD from 3 independent experiments with 5 replicates each one. Significant differences * *p* < 0.05, ** *p* < 0.01, *** *p* < 0.001, respect to the cells without treatment, according to one-way ANOVA and Tukey’s multiple comparison test (*p* < 0.05).

Figure 3 shows the levels of pro- and anti-inflammatory cytokines: TNF-α, IL-6, IL-2, IL-1β, IFN-γ, IL-12, IL-4 and IL-10 present in the culture supernatant of F3II cells treated in vitro with ¼ IC_50_, ½ IC_50_ and the IC_50_ of *H. junceus* scorpion venom. At 24 h of incubation (Figure 3A), the concentration of TNF-α increased significantly (*p* < 0.05) with the IC_50_ of *H. junceus* scorpion venom, while the treatment with ½ IC_50_ and the IC_50_ of the venom significantly decreased the concentration of IL-6 (*p* < 0.001). However, 48 h after treatment with the venom, statistically significant differences were observed in four of the eight evaluated cytokines with respect to cells treated only with culture medium. In this case, treatment with ¼ IC_50_ of the venom significantly decreased the concentration of IFN-γ (*p* < 0.001), while ½ IC_50_ induced a significant decrease in IL-1β levels (*p* < 0.01) and a significant increase in IL-12 concentration (*p* < 0.001). Finally, after 48 h of incubation (Figure 3B), the IC_50_ of the venom of *H. junceus* scorpion venom significantly decreased (*p* < 0.001) the levels of IL-1β and IFN-γ, while significantly increasing (*p* < 0.05) the concentration of TNF-α present in the F3II culture supernatant.

### 3.4. Determination of Cytokine Levels in Sera and F3II Tumor Lysates

**Figure 4:**
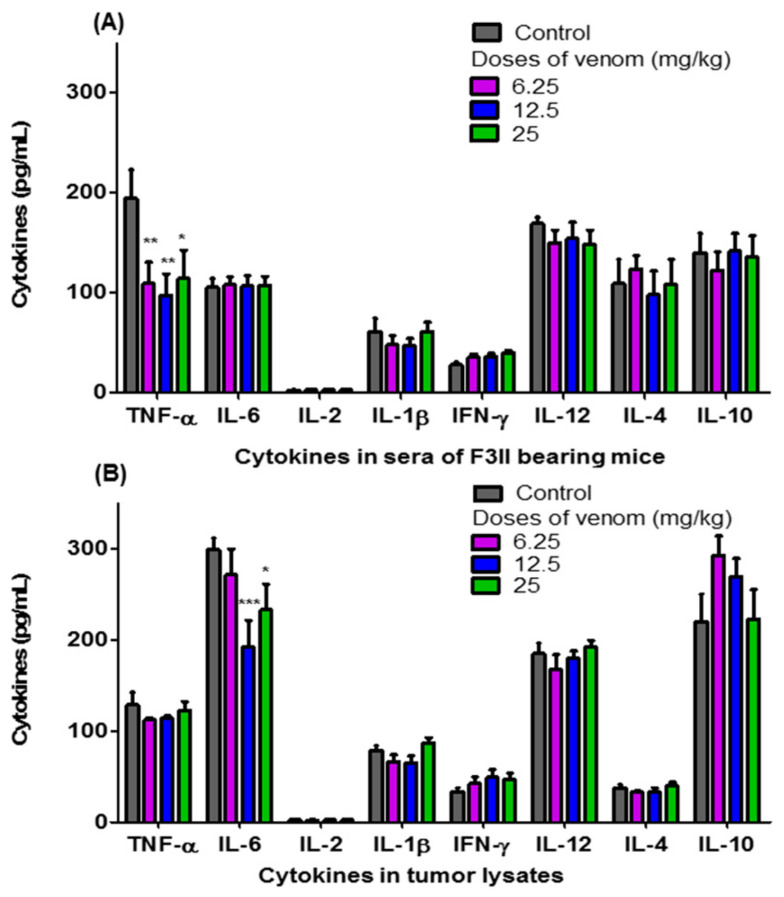
Levels of pro- and anti-inflammatory cytokines present in sera of F3II bearing mice and tumor lysates. For 24 consecutive days, 6.25 mg/kg, 12.5 mg/kg and 25 mg/kg of the scorpion venom *H. junceus* were orally administered to mice implanted with the F3II metastatic breast tumor. Twenty-four hours after the last dose, blood was drawn, sera were obtained, and the tumors were removed and macerated to assess the levels of proinflammatory and anti-inflammatory cytokines, using a commercial kit (BD OptEIA™; BD Biosciences, San Diego, CA, USA). The graphics represent the mean ± SD of TNF-α, IL-6, IL-2, IL-1β, IL-12, IFN-γ, IL-10 and IL-4 present in sera (**A**) and in 100 μg/mL of the tumor lysate (**B**). The meta-analysis of 3 experiments were carried out with seven animals each one is represented. Significant differences * *p* < 0.05, ** *p* < 0.01, *** *p* < 0.001, respect to untreated group with scorpion venom, according to one-way ANOVA and Tukey’s multiple comparison test (*p* < 0.05).

Figure 4 shows the levels of TNF-α, IL-6, IL-2, IL-1β, IFN-γ, IL-12, IL-4, and IL-10 in the sera of F3II bearing BALB/c mice treated orally with different doses of *H. junceus* scorpion venom (Figure 4A), as well as those in tumor lysates (Figure 4B). A significant decrease in the levels of TNF-α present in the sera of BALB/c mice implanted with the F3II tumor was observed when treated with 6.25 (*p* < 0.01); 12.5 (*p* < 0.01) and 25 mg/kg (*p* < 0.05) of *H. junceus* scorpion venom (Figure 4A). No statistically significant differences (*p* > 0.05) were observed between the levels of IL-6, IL-2, IL-1β, IFN-γ, IL-12, IL-4 and IL-10 present in the sera of tumor-bearing mice treated with the venom compared to the untreated control group (Figure 4A). Meanwhile, of all the cytokines evaluated in the F3II tumor lysates, IL-6 was the only one that showed a significant decrease in tumor lysates in the groups treated with 12.5 mg/kg (*p* < 0.001) and 25 mg/kg (*p* < 0.05) of *H. junceus* scorpion venom (Figure 4B).

## 4. Discussion

Breast cancer is one of the most commonly diagnosed cancer types, with an estimated 2.3 million new cases and 685,000 deaths in 2020 [23]. In the present study, the antitumoral effect in vivo of *H. junceus* scorpion venom by oral route in F3II murine mammary adenocarcinoma was evaluated for the first time. It was shown that oral administration of the venom 28 days after tumor implantation, a significantly decreases (*p* < 0.05) of tumor progression at 12.5 and 25 mg/kg was observed. These results were also demonstrated by showing a significant decrease (*p* < 0.05) in the weight of the tumor at these doses of venom. It is important to highlight that the F3II tumor line is a highly invasive and metastatic variant originating from a clone of a breast tumor developed spontaneously in BALB/c mice, widely used in preclinical research of drugs with antitumor potential [24].

Assessment of oral short term, subchronic toxicity, and teratogenic effect showed that low or moderate dose of *H. junceus* scorpion venom by oral route did not affect the health of the animals and has low impact on reproductive physiology in NMRI mice [25]. It is the only route that has yielded positive results when used to treat patients for malignant neoplasms, specifically as natural and traditional medicine. The present study suggests that active components of scorpion venom can reach the tumor at concentrations that delay its growth when administered orally. Previous pharmacokinetic studies carried out with the *H. junceus* venom by the oral route have shown its bioavailability is low, which suggests high degradation of venom components while maintaining the tumor-targeting properties [26].

Other in vivo studies with scorpion venoms in tumor-bearing animals have been performed, such as treatment with 17.5, 35, 52.5 μg topical twice a week for 16 weeks with whole venom of *Leiurus quinquestriatus* Hemprich & Ehrenberg, 1829, that decreased skin carcinogenesis incidence in mice [27]. Additionally, the administration of *Androctonus amoreuxi* whole venom intraperitoneally with 0.22 mg/kg/day for 13 days (20% LD dose) [28]. The 20% LD of the venom in this study taken orally would be around 400 mg/kg and we used doses lower than this value. Furthermore, none of the previous in vivo studies with other scorpion venoms use the oral route of administration.

The most abundant components of this venom present molecular weights around 4 kDa, known to be K^+^-channel specific peptides, and 7 kDa, known to be Na^+^-channel specific peptides; and this venom is rich in peptides that have of the same molecular masses of the peptides purified from other scorpions that affect ion-channel functions [20]. The antitumor and antimetastatic effect exerted by the modulation of the physiological activity of Na^+^ and K^+^ ion channels related to cell proliferation, migration and metastasis could be studied.

The effect of F3II tumor lysates from mice treated with the scorpion venom *H. junceus* was evaluated. First, it was observed that the lysate significantly decreased the viability of F3II for the lysates from the groups treated orally with 12.5 and 25 mg/kg of venom. These results constitute the first experimental evidence of the effect of tumor lysates on the viability of tumor cells and suggest that this scorpion venom could modulate intratumoral biomolecules that may contribute to its antitumor effect.

One goal of the present study is to evaluate the effect of *H. junceus* scorpion venom on the levels of pro and anti-inflammatory cytokines that could be involved in the antitumor effect observed in vitro and in vivo in F3II breast adenocarcinoma. Cytokines are pleiotropic molecules that, depending on the concentration and type of cells that stimulate their release, can have a pro and antitumor effect [29]. For this reason, the effect of *H. junceus* scorpion venom on the release of cancer-associated cytokines in the F3II tumor model was determined for the first time. Tumor cells secrete cytokines such as VEGF, TGF-β, IL-6, and Macrophage Colony-Stimulating Factor (MCSF), which increase angiogenesis, promote suppressor cell migration to the tumor site and inhibit the specific CTL response [30]. Currently, there is evidence that inflammatory cytokines and chemokines such as TNFα, IL-1β, and IL-6 may be produced by tumor cells and/or tumor-associated leukocytes and platelets and may directly contribute to malignant progression [31].

In the F3II culture supernatant, a significant reduction (*p* < 0.05) was observed in the IL-6 concentration compared to the untreated group after 24 h of treatment with the venom, which is positive in the analysis of the efficacy of venom as an antitumor treatment. Previous works have described that the overexpression of IL-6 in tumor lines, such as MCF-7, induces the epithelial-mesenchymal transition and increases its invasiveness [32]. Therefore, the demonstrated decrease in IL-6 levels could justify the significant reduction in the viability of the F3II tumor line treated with the venom compared to the untreated control, which constitutes an unprecedented study for scorpion venoms.

Related to TNF-α, the IC_50_ of *H. junceus* scorpion venom increased its levels in the culture supernatant of F3II cells treated for 24 and 48 h. TNF-α can have a pro- or antitumor effect depending on whether it is secreted by the tumor microenvironment or by the tumor cells themselves [33], so we suggest that the modulation of this cytokine by scorpion venom *H. junceus* favors its antitumor effect. Another cytokine that increased significantly (*p* < 0.001) compared to the untreated control in the F3II supernatant was IL-12, but this time after treatment for 48 h of incubation with ½ IC_50_, which is a cytokine involved in the effect antitumor and anti-metastatic of natural products such as curcumin [34].

A pro-inflammatory and pro-tumor cytokine that decreased its concentration after treatment with ½ IC_50_ and the IC_50_ of *H. junceus* was IL-1β, which was significantly observed after 48 h of incubation. Within a tumor, IL-1β is produced and secreted by various types of cells such as those of the immune system, fibroblasts, or tumor cells. In cancer, IL-1β has a pleiotropic effect on cells of the immune system, angiogenesis, proliferation, migration, and metastasis of tumor cells [35], so its decrease promoted by venom may contribute to its antitumor effect. However, future studies need to evaluate the ability of the *H. junceus* scorpion venom to modulate all cell populations, as well as determine the intracellular concentration of cytokines.

Treatment of F3II breast tumor with ¼ IC_50_, ½ IC_50_, and the IC_50_ of *H. junceus* scorpion venom decreased IFN-γ levels after 16 h of treatment, which was also observed at 48 h for ¼ IC_50_ and the IC_50_ of the venom. Despite the fact that many investigations point to the antitumor potential of IFN-γ, it has been shown that this cytokine is capable of promoting inflammatory cellular and molecular mechanisms that promote tumor initiation, immunoevasion, and tumor cell survival [36]. The protumoral functions of IFN-γ depend on the type of tumor, microenvironment factors, and signal intensity [34]. In addition, IFN-γ has been associated with maintaining the growth of invasive ductal carcinoma of the breast, so reducing its levels in this particular case would be favorable, but not at the systemic level because the secreted CTL could destroy tumor cells [37].

*Heteroctenus junceus* scorpion venom, depending on the concentration and incubation time, promotes a decrease in the cytokines IL-6, IL-1β and IFN-γ; as well as an increase in TNF-α and IL-12 in the supernatant of the F3II tumor cells. These results constitute the first experimental evidence on this subject for this scorpion venom using in vitro models, which was also evaluated in the murine model implanted with this tumor.

In the studies carried out on the sera of animals with F3II tumors treated with the scorpion venom, it was shown that seven evaluated cytokines (IL-6, IL-2, IFN-γ, IL-1β, IL-12, IL-4, and IL-10), displayed same concentration (*p* > 0.05) respect to the untreated control. However, the three doses of *H. junceus* scorpion venom tested significantly decreased (*p* < 0.05) the levels of TNF-α in the serum of BALB/c mice implanted with the F3II tumor. Al-Asmari et al. (2016) [38] and Kampo et al. (2019) [39] documented that the venoms from *Leiurus quinquestriatus* Hemprich & Ehrenberg and *Buthus martensi* Karch scorpions, respectively, have an immunomodulatory effect on inflammatory mediators such as NF-κB, TNF-α and IL-6; while He et al. (2021) reported that in vitro activation of TNF-α induces invasive and malignant behavior in breast tumor cells [40]. The decrease in TNF-α levels in sera of animals with a tumor and treated with *H. junceus* scorpion venom could be due to the fact that previous results suggest that this venom is capable of blocking voltage-gated sodium channels 1,5 [41]. This type of ion channel is overexpressed in breast tumors, which is associated with tumor progression and its blockade by the venom of the *Buthus martensi* scorpion has been shown to induce a reduction in the activation of NF-κB and TNF-α [39].

IL-6 was the only cytokine in the tumor lysate that significantly changed its concentration (*p* < 0.05) after treatment with 6.25; 12.5 and 25 mg/kg of *H. junceus* scorpion venom, compared to the untreated group. The significant decrease observed in the levels of IL-6 in the tumor lysate is of great importance and could explain the effect on the reduction in tumor volume induced by *H. junceus* scorpion venom on F3II, since this cytokine promotes the proliferation of tumor cells, migration, invasion, angiogenesis, and metastasis [42]. In addition, the greatest significant reduction in IL-6 (*p* < 0.05) was observed at the 12.5 mg/kg dose, which was where the greatest reduction in F3II tumor volume was achieved. This involvement of IL-6 in the in vivo antitumor effect of a natural product coincides with what was observed after oral administration of 50 mg/kg of curcumin for 20 days to C57BL/6 mice implanted with Lewis lung carcinoma [43].

The movement in the aforementioned cytokines creates a microenvironment that could contribute to tumor control, in parallel to the direct antitumor effect on tumor cells, previously demonstrated for the venom studied. On the other hand, considering that all the cytokines were evaluated at a certain time (24 h) and that they have different kinetics in the organism due to the fact that their expression is not simultaneous, it is necessary to evaluate their behavior after administration of *H. junceus* scorpion venom at different times in future studies. However, previous studies have shown that there were no changes in pro-and anti-inflammatory cytokine levels, at 2, 4, 6, 8, and 24 h after the last administration of ten doses of 3.2 mg/kg of *H. junceus* by intraperitoneal route [44].

## 5. Conclusions

*Heteroctenus junceus* scorpion venom by oral route can inhibit the mammary tumor progression. The IL-6 cytokine could be the main soluble mediator involved in the inflammation associated with cancer, modulated by *H. junceus* scorpion venom. However, depending on the experimental conditions, it is capable of decreasing the release of other protumoral cytokines such as IL-1β and IFN-γ as well as increasing the antitumor cytokines TNF-α and IL-12. These results confirmed that this scorpion venom can modulate the concentration of pro-inflammatory cytokines in F3II tumor cells, which could explain the antitumor observed effect in animal model and could be an attractive natural product for developing further therapeutic agent against breast cancer.

## Data Availability

The data presented in this study are available in article and supplementary material.

## Supplementary Materials

The following supporting information can be downloaded at: https://www.mdpi.com/article/10.3390/life13122287/s1, Figure S1: HPLC separation of soluble venom; Table S1: Partial de novo sequencing derived from MS/MS of integral *H. junceus* scorpion toxin precursor ions.
