# Supplementary material for: Heteroctenus junceus Scorpion Venom Modulates the Concentration of Pro-Inflammatory Cytokines in F3II Tumor Cells
Source: Life (Basel). 2023 Nov 30;13(12):2287. doi: 10.3390/life13122287 (PMC10871110; doi:10.3390/life13122287)
Supplement: Supplementary file 1 [file life-13-02287-s001.zip › life-2478872-supplementary.pdf]

Supplementary Materials:

Biochemical and molecular characterization of venom from *Heteroctenus junceus* scorpions maintained in the laboratories of The Entrepreneurial Group of Biopharmaceuticals and Chemistry Productions (LABIOFAM, Havana, Cuba). Venom was obtained using electrical stimulation, which was dissolved in double distilled water and centrifuged at 10 000 *g* for 15 min. The supernatant was filtered using 0.2 μm syringe filters and stored at -20 °C until used.

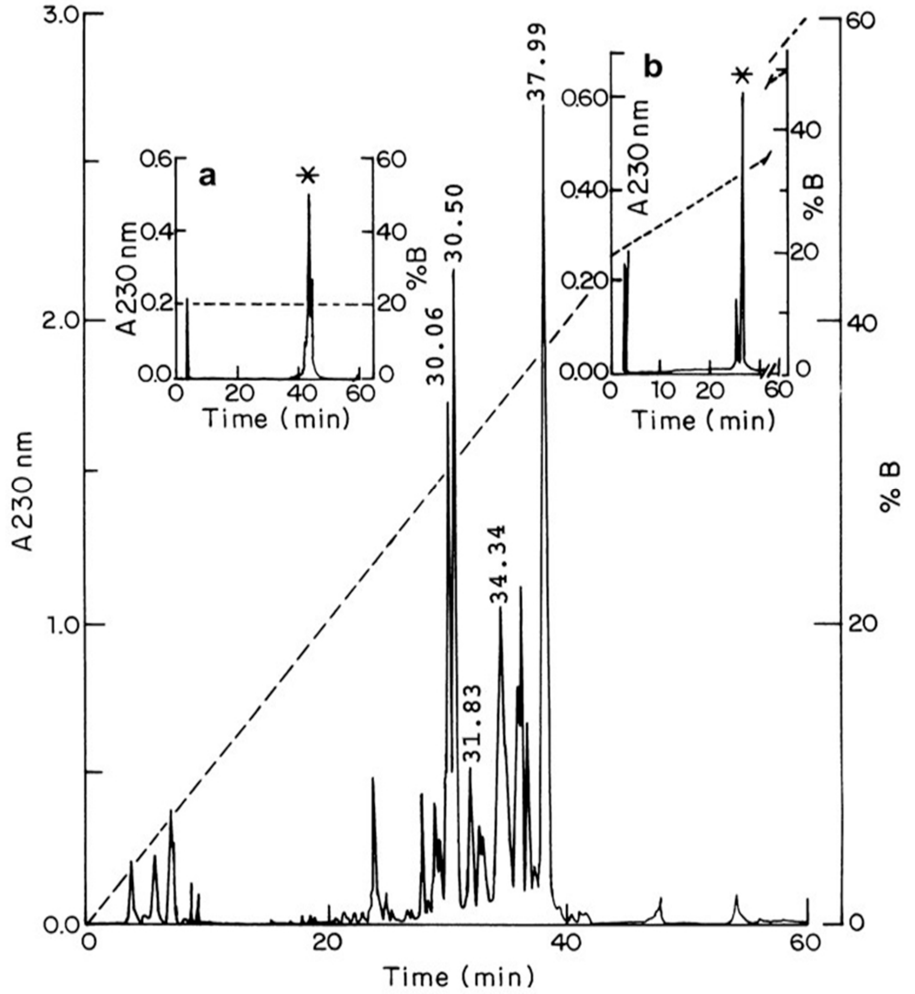

Figure S1. HPLC separation of soluble venom. Soluble venom (1 mg) was applied to a C18 reverse-phase column and resolved using a gradient from solution A to 60 %solution B, during 60 min (see material and methods). Fractions eluting at 30.06, 30.50, 31.83, 34.34 and 37.99 min corresponded respectively to 8.1, 14.5, 3.5, 10.5 and 27.3 % of the material recovered. Insert shows the profile of further separation of fraction eluting at 34.34 min, using a C18 reverse-phase column run for 60 min with constant concentration of 20 % solution A. From a sample containing 180 mg applied to the column, the peptide indicated by the asterisk corresponded to 54 % of the material recovered. Insert b shows HPLC profile of further separation of 80 mg of component that elutes at 37.99 min using a gradient from 20 % to 50 % solution B, during 60 min. Component labeled with asterisk was the main component and corresponded to 84 % of the material recovered (Taken and modified of García-Gómez et al., 2011 [19]).

Table S1: Partial de novo sequencing derived from MS/MS of integral *H. junceus* scorpion toxin precursor ions (Taken and modified of Rodríguez-Ravelo et al., 2015 [20]).

HPLC-RT (min)	MW (Da)	Precursor ion	m/z	*De novo* sequence
21.20	4007.84	668.98	6+	…KAVGSLQSKC…
21.98	3876.00	776.20	5+	VFLNVKLCKGAA…
21.98	4059.12	1015.78	4+	…TLLVKCTSSKL…
23.02	3072.54	769.14	4+	…APPAAEAMGAKCGNKLME…
23.02	4039.78	808.95	5+	…TPGCYCKSKKCLGMT…
23.17	3622.57	1812.29	2+	…VDENSFDCYLL…
23.45	3949.95	1317.65	3+	…TVKCTSPKQCVPACKAAMG …
25.15	1922.88	961.95	2+	…QELVG… e HexNAc eHexNAc
26.03	3273.44	1637.22	2+	(HexNAc)4-(DeoxyHex)
27.19	6549.01	1092.34	6+	…TFLYGGVGGN…
27.42	4688.12	1013.96	4+	…VKKCFSSSEC…
27.74	3100.56	776.40	4+	…EAAGAMGAKACNKLME…
27.74	7088.13	1013.46	7+	…LNWSCYCEGLPDDS…
28.43	7095.06	1183.51	6+	…LPENAEVWDSST…
28.83	7093.18	1183.21	6+	…KLNWCYCEGLPDNS…
29.23	7273.41	1213.40	6+	…LKLSCWCTGLPD…
29.23	7324.33	1047.34	7+	…NSCYCYGLPE…
29.73	8067.65	1345.78	6+	…NLPENAEVWDSST…
31.07	1523.70	762.85	2+	…DELDKSGFGG…
31.07	7263.20	1211.53	6+	…GYCYSPGMSCYCTNLPED…
31.95	7041.12	1174.52	6+	…CYSFGLSCYCTNL…
32.55	7044.50	1410.60	5+	…TLGLSCYCTGLPEDAKV…
32.55	7060.98	1177.52	6+	KDLGYPVDWGN…
32.96	7437.51	1063.51	7+	…TGYCYFWKLACYCEGLPN…
33.76	7424.52	1238.42	6+	…TGYCYFWKLACYCEGLPN…
34.06	7318.12	1220.69	6+	…GTLLWGDPGTGPC…
35.09	6853.02	1143.17	6+	…ACYCEGLPDDV…
35.09	7126.08	1156.34	6+	…VNTEYSWA…
36.45	7954.59	1137.37	7+	…NLPENAEVWDP…
36.96	7287.18	1215.36	6+	…NLPDNAEVWDS…
37.51	7280.18	1214.35	6+	…LPNDAQVWDSS…
